# Linking robust spatiotemporal datasets to assess and monitor habitat attributes of a threatened species

**DOI:** 10.1371/journal.pone.0265175

**Published:** 2022-03-17

**Authors:** Chris Witt, Raymond J. Davis, Zhiqiang Yang, Joseph L. Ganey, R. J. Gutiérrez, Sean Healey, Shaula Hedwall, Serra Hoagland, Ron Maes, Karl Malcolm, Jamie Sanderlin, Mark Seamans, Gavin M. Jones

**Affiliations:** 1 USDA Forest Service, Rocky Mountain Research Station, Boise, ID, United States of America; 2 USDA Forest Service, Pacific Northwest Region, Corvallis, OR, United States of America; 3 USDA Forest Service, Rocky Mountain Research Station, Ogden, UT, United States of America; 4 USDA Forest Service, Rocky Mountain Research Station, Flagstaff, AZ, United States of America; 5 Dept. of Fisheries, Wildlife, and Conservation Biology, University of Minnesota, St. Paul, MN, United States of America; 6 US Fish and Wildlife Service, Arizona Fish & Wildlife Conservation Office, Flagstaff, AZ, United States of America; 7 USDA Forest Service, Rocky Mountain Research Station, Missoula, MT, United States of America; 8 USDA Forest Service, Southwestern Region, Albuquerque, NM, United States of America; 9 US Fish and Wildlife Service, Division of Migratory Bird Management, Lakewood, CO, United States of America; 10 USDA Forest Service, Rocky Mountain Research Station, Albuquerque, NM, United States of America; Southeastern Louisiana University, UNITED STATES

## Abstract

Accessibility of multispectral, multitemporal imagery combined with recent advances in cloud computing and machine learning approaches have enhanced our ability to model habitat characteristics across broad spatial and temporal scales. We integrated a large dataset of known nest and roost sites of a threatened species, the Mexican spotted owl (*Strix occidentalis lucida*), in the southwestern USA with Landsat imagery processed using the Continuous Change Detection and Classification (CCDC) time series algorithm on Google Earth Engine. We then used maximum entropy modeling (Maxent) to classify the landscape into four ‘spectral similarity’ classes that reflected the degree to which 30-m pixels contained a multispectral signature similar to that found at known owl nest/roost sites and mapped spectral similarity classes from 1986–2020. For map interpretation, we used nationally consistent forest inventory data to evaluate the structural and compositional characteristics of each spectral similarity class. We found a monotonic increase of structural characteristics typically associated with owl nesting and roosting over classes of increasing similarity, with the ‘very similar’ class meeting or exceeding published minimum desired management conditions for owl nesting and roosting. We also found an increased rate of loss of forest vegetation typical of owl nesting and roosting since the beginning of the 21^st^ century that can be partly attributed to increased frequency and extent of large (≥400 ha) wildfires. This loss resulted in a 38% reduction over the 35-year study period in forest vegetation most similar to that used for owl nesting and roosting. Our modelling approach using cloud computing with time series of Landsat imagery provided a cost-effective tool for landscape-scale, multidecadal monitoring of vegetative components of a threatened species’ habitat. Our approach could be used to monitor trends in the vegetation favored by any other species, provided that high-quality location data such as we presented here are available.

## Introduction

Assessing and monitoring habitat across large spatial and temporal scales is a recurring challenge for natural resource managers. Long-term monitoring efforts that cover large geographical extents are difficult because logistics, cost, localized data, outdated data, and inconsistent data collection add constraints to such endeavors [1–3]. The difficulties of monitoring wildlife habitat across broad scales are even more pronounced for rare species or those with small or narrowly defined ecological niches [4]. As land managers are challenged to apply the best scientific data available to properly guide and evaluate management strategies, there is a need for procedures to assess and monitor species habitat at low cost and at the appropriate spatial scales and temporal frequencies.

The use of remotely sensed data to inform natural resource management at broad spatial and temporal scales has increased considerably in recent times [5–7]. Landsat imagery is collected through a series of Earth observing satellites by NASA and USGS with nearly 50 years of continuous observation [8]. The Landsat series of satellites measure reflectance at wavelengths and ground resolutions appropriate for monitoring forest cover trends, and Landsat imagery has been distributed free of charge since 2008. This has facilitated its use in ecological applications [9], as has the development of cloud-based platforms capable of processing large areas quickly. Landsat data have proven useful in identifying vegetation conditions associated with habitat of avian species of interest [10, 11]. However, Landsat signal variation caused by both systematic seasonal factors and more ephemeral atmospheric factors in early efforts has limited applicability across multiple time periods. The recent application of time series analysis allows researchers to identify and use the central tendency of Landsat reflectance over time to minimize the effect of such issues [5, 12]. Emerging applications for temporally stable, accurate landscape-scale habitat maps include long-term wildlife habitat monitoring programs [13, 14] and species recovery efforts [15].

Here, we used multispectral, multitemporal imagery to model and map trends in vegetation associated with an iconic and threatened wildlife species, the Mexican spotted owl (*Strix occidentalis lucida*) across a large portion of the species’ distribution in southwestern North America. We focused our modeling on the component of owl habitat that forest managers can affect—forest vegetation that owls use for nesting and roosting. Mexican spotted owls (“owls” hereafter) often live in mature, seasonally dry forests where fire can be an integral part of the ecosystem [15]. The owl was listed by the U.S. Fish and Wildlife Service (FWS) in 1993 as “threatened” under the Endangered Species Act [16] due to perceived reductions of its habitat over the past century. When the species was listed, the FWS identified three reasons for the owl’s decline: (i) past logging that resulted in the loss of nesting/roosting forest structure and the promotion of even-aged stand structure (thus reducing understory and landscape heterogeneity); (ii) the future threat of these practices; and (iii) the potential loss of habitat owing to the effects of high-severity, stand-replacing wildfires.

Mature mixed-conifer and pine-oak (*Pinus* spp.–*Quercus* spp.) forests provide the majority of breeding habitat for owls in forested landscapes [17–19]. These forests provide high canopy cover, high density of large trees, and complex forest structure used by the owl for nesting and roosting. However, past land management (of both forest and rangelands) and fire suppression have altered the structural complexity of these forest types [20–22]. Therefore, vegetation composition and structure are important components of owl habitat that are of high interest to resource managers because they are the components most typically modified by management actions. The 2012 Mexican Spotted Owl Recovery Plan explicitly states two general criteria to be met before the owl can be delisted. The first involves trends and status of owl occupancy rates, but the second pertains to the stability and trend of the key habitat variables for suitable nesting and roosting referenced above [15]. Use of the methodology described in this study may assist with determining the degree of progress made toward meeting this second criterion across much of the owl’s range. To this end, we integrated Landsat imagery with known owl nest and roost locations and a broad-scale, standardized, ground-based vegetation dataset to: (i) develop a predictive model for owl-associated vegetation types across Arizona and New Mexico from 1986 to 2020; (ii) quantify forest characteristics of modeled owl-associated vegetation using extensive inventory data; and (iii) summarize changes in the regional distribution of owl-associated vegetation.

## Materials and methods

### Study area

**Fig 1:**
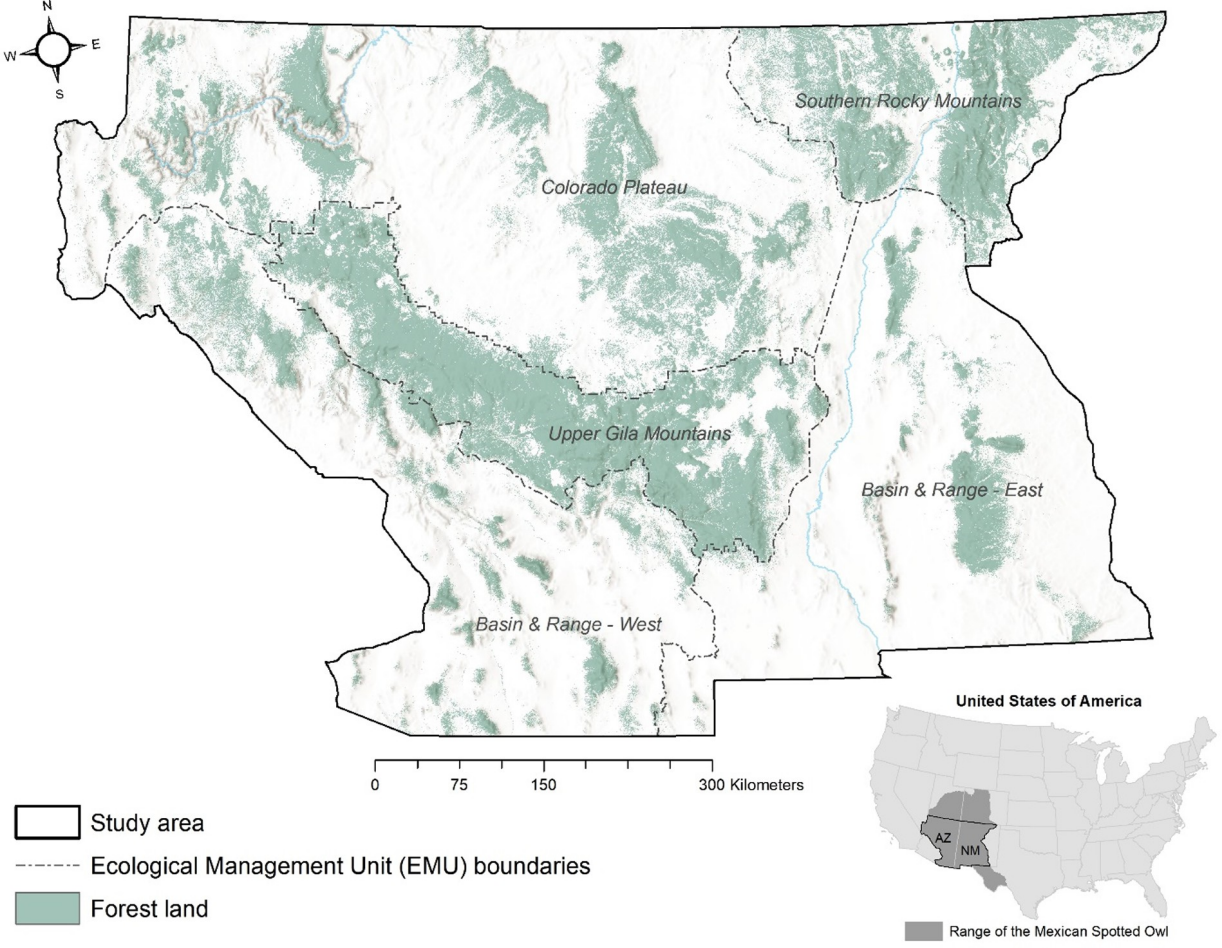
Study area map. Map shows the study area in Arizona and New Mexico, displaying boundaries of Ecological Management Units defined in USFWS (2012) and the extent of forested lands (National Land Cover Database). Inset shows the location of the study area relative to the range of the Mexican spotted owl within the U.S.

Our study area covered 457,000 km^2^ containing about 119,000 km^2^ of forested land (as defined by the U.S. Forest Service’s Forest Inventory and Analysis [FIA] program; [23]) in Arizona and New Mexico, roughly the central portion of the owl’s range in the southwestern U.S. (Fig 1). We restricted the study area to Arizona and New Mexico for several reasons: (i) our work represented a cooperative effort with the U.S. Department of Agriculture, Forest Service, Southwestern Region, which encompasses Arizona and New Mexico; (ii) our available owl nest and roost location data were within this area; (iii) the northern part of the owl’s range is often in the sparsely vegetated cliff and canyon landscapes of Utah and Colorado that are relatively less subject to forest management and where our focus on the vegetative component of owl habitat might be less applicable (although future study is warranted); and (iv) little or no FIA or nest site data was available for the southern portion of the owl’s range in Mexico.

Forest communities in our study area range from mesquite (*Prosopis* spp.) and pinyon-juniper (primarily the *P*. *edulis-Juniperus monosperma* association) woodlands at the lower elevations, ponderosa pine (*P*. *ponderosa*), oak, and dry mixed-conifer (commonly co-dominated by Douglas-fir [*Pseudotsuga menziesii*] and ponderosa pine) communities at mid-elevations, and more mesic mixed-conifer (dominated by white fir, *Abies concolor*) and spruce-fir (primarily *Picea engelmannii-Abies lasiocarpa*) at the higher elevations. The study area covered five ecological management units (hereafter “EMUs”, [15]). EMUs were based on the geographical subdivisions of the owl’s range that the U.S. Fish and Wildlife Service has used to organize owl recovery efforts. The Mexican Spotted Owl Recovery Plan ([15] hereafter “Recovery Plan”) based these EMUs on existing delineations of physiographic provinces, biotic regimes, threats to the owl, and administrative boundaries; distribution of discrete owl populations was a minor factor in this delineation as owls move between EMUs [15]. We did not consider areas outside of these EMUs in our analyses.

### Owl nest/roost sites

To facilitate model development, we compiled a database of known Mexican spotted owl nest/roost locations recorded from 1989 to 2020 on public lands in Arizona and New Mexico. Our database included nest/roost sites from eight independent data sets, including previous demographic studies [24], species recovery planning efforts [25–28], US Forest Service project-level surveys, and opportunistic nest/roost observations (see S1 Table).

We began with a raw database of all nest/roost locations and applied decision rules for quality control that yielded a refined database of high-quality locations. The raw database contained 7,455 nest/roost locations across the study area. We applied five decision rules for quality control to these data sets to maximize data quality and standardization, reduce potential effects of pseudoreplication, and reduce the likelihood that observations might represent behaviors other than nesting or roosting. First, we eliminated roost location(s) when surveyors detected a nest location during the same survey. This situation arose frequently in data originating from two of the demographic studies [24] because one member of the owl pair or young were detected on the nest while another owl was detected roosting elsewhere; we considered nest locations to be more biologically important than roost locations. Second, we eliminated duplicate roost locations when both individuals of a pair were detected and recorded at the same location, so that only a single record was used from that location. Third, we selected only observations that occurred during the height of the breeding season, which we conservatively defined as 1 March through 31 July. Observations after this period are less likely to represent breeding habitats because owls tend to expand their space use outside the main breeding season [22, 29]. Fourth, we selected only one nest location per territory per year. Multiple within-year nest locations could be the result of recording inaccuracies due to map scale. If two locations existed, we selected the earlier one because early season locations are more likely to be determined from direct observations of the adult or nestlings. If more than two locations existed, we selected the most frequently recorded nest location. Fifth, we eliminated any detections recorded as roosts that occurred during twilight or nocturnal hours (adjusted by geographic location within the region), because owls may have already moved from their day roost by the time surveyors recorded these detections [29–31].

Applying our decision rules reduced the original raw data set of 7,455 nest/roost locations to a refined data set of 2,913 nest/roost locations. We then re-projected all locations to the WGS84 coordinate system for compatibility with available high-resolution satellite imagery and performed visual inspection of all locations overlaid on that imagery to ensure locations were not mapped in obvious non-forested areas (e.g., grasslands) because of data recording errors. Finally, because we modeled predictor variables (see below) using Landsat imagery with a spatial grain of 30m pixels, all annual locations within 30m of another were dissolved into one representative 30m pixel location. The final modeling dataset included 2,233 nest/roost locations.

### Modeling owl vegetation similarity

We applied the Continuous Change Detection and Classification (CCDC) time series algorithm [5] to all imagery from Landsat 5, 7, and 8 from 1986 to 2020 on the Google Earth Engine platform [32]. CCDC fits harmonic functions to all cloud-free images for each reflectance band on a per-pixel basis. This process stabilizes the time series of covariates to reduce annual variation to better identify and interpret real changes. CCDC implements an effective break-finding process that identifies points in time where detectable change has occurred [33]. New harmonic functions are fitted after these breaks.

We produced 54 Landsat metrics for each pixel within the study area for each year. These included eight harmonic parameters describing the time series function and rooted mean squared error for the harmonic fitting for each of six Landsat spectral bands. These CCDC-based metrics were extracted for each owl location corresponding to August 1^st^ of the year of the field observation; this mid-growing season date assured leaf-on signal of deciduous trees in the region. The model described below was calibrated using 54 Landsat metrics corresponding to the year of field observation but was then applied to the set of Landsat metrics for each year, creating a time series of habitat similarity maps. This approach, which relies upon the defensible [34] assumption of consistent Landsat radiometry from year to year, has been applied elsewhere [35, 36].

We used the open source program Maxent [37] to produce 10 bootstrapped replicate models using training data comprised of a random selection of 50% of the 2,233 owl nest/roost locations (thinned from 7,455 sites as described above) for each replicate. The remaining 50% were reserved for assessing model performance. We matched field observed year to the spectral properties used for that year and built the model across years using all the observations. We evaluated model performance using the area under the curve (AUC) statistic [38] and another threshold-independent evaluator called the continuous Boyce index (CBI) which is based on the Spearman rank correlation of a model’s predicted-to-expected (P/E) ratio curve [39]. In our case, the P/E ratio is the proportion of held-out test pixels to the proportion of available forest-capable pixels calculated along the entire spectrum of the model’s predictive index from low to high (0 to 1). Forest-capable pixels were those mapped with tree cover in any of seven national land cover maps circa 2001–2016, available from the National Land Cover Database [40]. The P/E ratio curve was produced using a moving average (width = 0.1) of the P/E ratios for every 0.01 interval along the model’s predictive index from low to high (0 to 1). A P/E ratio of one (P/E = 1) indicates the proportion of held-out owl locations (test pixels) occurred in equivalent proportion to randomly selected background pixels, thus a random selection. A P/E ratio less than one (P/E<1) indicates that test locations occurred less than expected by chance, which we interpreted as avoidance by owls. A P/E ratio greater than one (P/E>1) indicates test locations occurred more than expected by chance, which we interpreted as selection by owls for nesting/roosting.

We used the Maxent regularization multiplier parameter [41] from 0.1 to 2.0 with steps of 0.1 to calibrate the Maxent model [13, 14]. We contrasted the owl locations (pixels) used for model training against a random sample of 10,000 background forest-capable pixels, drawn from the area labeled “forestland” in Fig 1, for each replicate [42]. We used the mean logistic model output as a continuous index of spectral similarity associated with vegetation conditions at observed owl locations. A similarity index with a value near zero represented forest cover that was spectrally very different from forest cover used by nesting/roosting owls; likewise, an index value close to 1 was spectrally very similar to forest cover used by nesting/roosting owls. We reclassified this similarity index into four spectral similarity classes based on the shape of the model’s P/E curve [39]. Finally, we applied the final Maxent model and similarity classifications to every forest-capable pixel in the study area for every year between 1986 and 2020.

### Summarizing map classification using forest inventory data

We used existing FIA plot data from 2007 to 2018 to characterize forest structure and species composition within our map classes. The FIA sample design consists of a semi-systematic, probabilistic sample of forest ecosystems that enables stratified estimation of forest attributes such as forest land area, number of trees, disturbance history and extent, growth, and mortality. FIA plot locations have a spatial intensity of approximately one plot per 2,428 ha (6,000 ac) across all states, forest cover types, and ownerships [43]. Each plot consists of four 7.3m radius subplots, with one subplot centrally located and three others extending 36.6m from subplot center at 120-, 240-, and 360-degree azimuths. We used the summarized FIA data to determine how selected structural components of our mapped similarity classes compared to conditions considered suitable for those components of nesting and roosting habitat, as defined in [15] (Tables C2 and C3).

The metrics we used to describe forest structure within our similarity classes included total basal area (TBA) of all live trees ≥ 2.5 cm (1 in.) diameter (measured at breast height (dbh) or root collar (drc), depending on species), % of total basal area from trees 30.5–45.7 cm (12–18 in.), > 40.6 cm (16 in.), or > 45.7 cm (18 in.) in diameter (%BA_12-18_, %BA_>16_, and %BA_>18_, respectively), % canopy cover (%CC), and the number of large (> 45.7 cm (18 in.) diameter (TPA_large_)) trees per hectare ([15]; Table 1). We chose these attributes because they describe desired nesting and roosting conditions described in the Recovery Plan. To calculate plot canopy cover, point transects extending 7.62 m (25 ft.) were established in each cardinal direction from the center of each of the four subplots. Cover was recorded at 30.5 cm (12 in.) intervals, summed across subplot transects, then averaged across all four subplots to acquire canopy cover values for the plot. All data collection followed protocols described in the Interior West FIA Phase 2 Field Procedures Manual [44].

**Table 1:** Desired minimum conditions for six structural attributes of Mexican spotted owl nesting/roosting habitat.

Structural attribute	Minimum	Source
	desired condition	
Canopy cover	40–60%	Table C.2
Large (>45.6 cm dbh) tree density	30–37 trees/ha	Table C.3
Basal area (trees >2.5cm dbh)	25.3–33.3 m^2^/ha	Table C.3
Proportion of basal area	30%	Table C.3
consisting of 30.5–45.6 cm dbh trees		
Proportion of basal area	50%	Table C.2
consisting of trees >40.6 cm dbh		
Proportion of basal area	30%	Table C.3
consisting of trees >45.6 cm dbh		

From Tables C.2 and C.3 in the Mexican spotted owl Recovery Plan (USFWS 2012). Ranges indicate components where desired conditions varied among forest types and/or Ecological Management Units.

To explore species composition of our mapped classes, we calculated the contributions to tree basal area from the following groups: Douglas-fir and white fir, ponderosa pines (including *P*. *ponderosa*, *P*. *engelmannii*, *P*. *leiophylla*, and *P*. *arizonica*), pinyon pine (primarily *P*. *edulis*, *P*. *monophylla*, *P*. *discolor*, and *P*. *cembroides*), and juniper (primarily *J*. *utahensis*, *J*. *scopulorum*, *J*. *monosperma*, and *J*. *deppeana*). These species groups served as a coarse index to forest types. Douglas-fir and white fir typically dominated the more mesic mixed-conifer forest types in which most owls nest [15]. Species in the ponderosa pine group dominated the ponderosa pine and pine-oak forest types, which are often a component of more xeric mixed-conifer that also serve as nesting sites in some EMUs but to a lesser extent than mesic mixed-conifer across the owl’s range [17, 18]. Pinyon-juniper woodland typically lacked the vertical structure associated with nesting cover and was rarely used by nesting owls outside of canyon lands [15, 18, 45].

To co-date FIA plots with our time series maps, we calculated the mean continuous similarity index of a 3-by-3-pixel block (an area that encompasses the FIA plot design) for each map year. We then matched FIA plots with maps by ensuring the plot measurement year co-dated the map year. Next, we applied the similarity class thresholds to the mean index for every plot to group them into similarity classes across all map years. Finally, we calculated mean plot values with corresponding 95% confidence intervals for FIA attributes for each map similarity class. In addition, we used a Generalized Linear Mixed Model (GLMM) to fit stand attributes on similarity class. Where a gamma distribution for positive responses was assumed, we used a data-driven rescaling that maps the observed distribution on the closed interval (0,∞) to the half-closed interval [0,∞) [46]. For a beta distribution for zero to one responses, we used a similar data-driven rescaling that maps the responses from the open interval (0,1) to the closed interval [0,1] as described in ref. [47]. Plotted data revealed potential differences in variance among class levels, so we included a random heteroskedastic term in the models that estimate separate variances for each level of class. The error degrees of freedom were calculated using the Kenward-Roger method [48]. Multiple comparisons between classes were adjusted for family-wise error by using the Tukey-Kramer method [49]. GLMM analyses were performed using SAS PROC GLIMMIX and multiple comparisons were performed using SAS PROC PLM software (SAS *ver*. *9*.*4*, *SAS/STAT ver*. *15*.*2*).

### Tracking changes in vegetation similarity

We applied our Maxent model annually to map four similarity classes across the region for each year from 1986 to 2020 to produce a predicted time series of changes in vegetation. We also tracked overall transitions among vegetation similarity classes from the beginning (1986) to the end (2020) of the study period.

To interpret cover type changes caused by forest disturbance we used available fire databases, including the Monitoring Trends in Burn Severity (MTBS) database [50]. Reliable and complete spatial layers of other forest disturbance types (e.g., timber harvest, insects, disease) that covered all forest-capable lands were not available. We allowed for 1–2 years of delayed detection because of the timing of the image acquisition related to the timing of a wildfire. By differencing cover type maps within a wildfire perimeter that bracket an individual disturbance event (post-disturbance map–pre-disturbance map) we produced a “cover type difference” map that reflected the magnitude of change caused by the wildfire.

We illustrated the local-scale utility of our methodology for quantifying changes in cover type class owing to disturbance events using the Rodeo-Chediski Fire of 2002. This fire occurred within the Upper Gila Mountains EMU in central Arizona that impacted roughly 187,000 ha with varying severity among several different forest types, including those deemed important for owl nesting and roosting [51, 52].

## Results

### Modeling similarity to owl nest/roost sites

**Fig 2:**
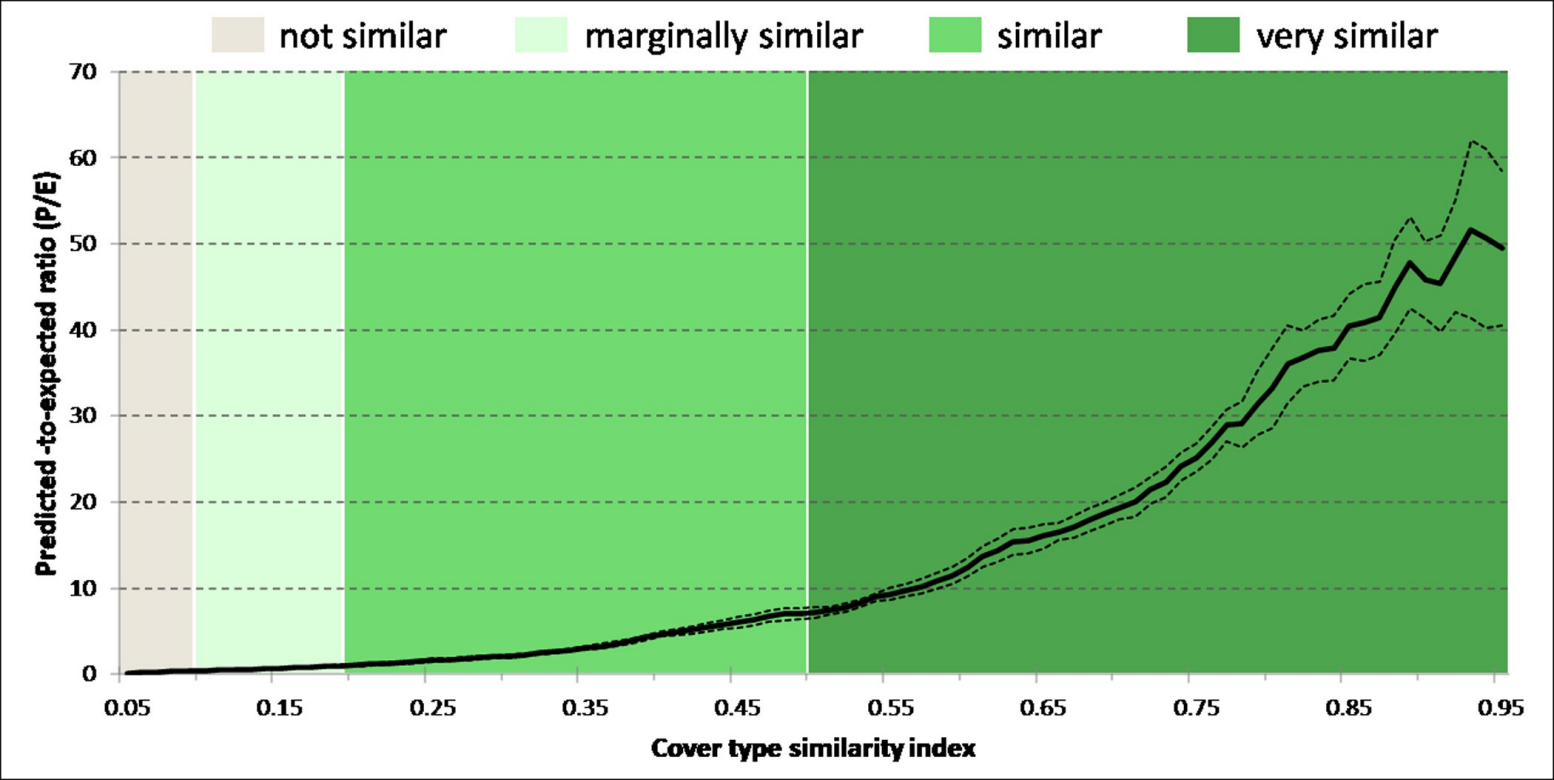
Predicted-to-expected (P/E) ratio curve. The P/E ratio curve [39] was used to evaluate, calibrate, and reclassify the forest vegetation cover type spectral similarity index model into four map classes. The solid black line is the mean P/E curve from bootstrapped replicates and dashed lines represent the 95% confidence intervals.

The forest vegetation spectral similarity outputs created by Maxent were stable across bootstrapped replicates and predicted owl use well (AUC_test_ = 0.939±0.001) producing a monotonically increasing P/E curve with a high positive Spearman rank correlation (CBI = 0.996±0.002). The reclassification of the continuous spectral similarity index based on the P/E curve (Fig 2) was as follows:

1. **Not similar (0–0.095):** Index values below the mean between zero and the P/E = 1 threshold.
2. **Marginally similar (0.096–0.191):** Index values above the mean between zero and the P/E = 1 threshold and below the P/E = 1 threshold.
3. **Similar (0.192–0.5):** Index values between the P/E = 1 threshold and the average index value at known owl locations.
4. **Very similar (0.51–1.0):** Values above the average owl location index value.

### Using forest inventory data to describe vegetation structure/composition of similarity classes

**Fig 3:**
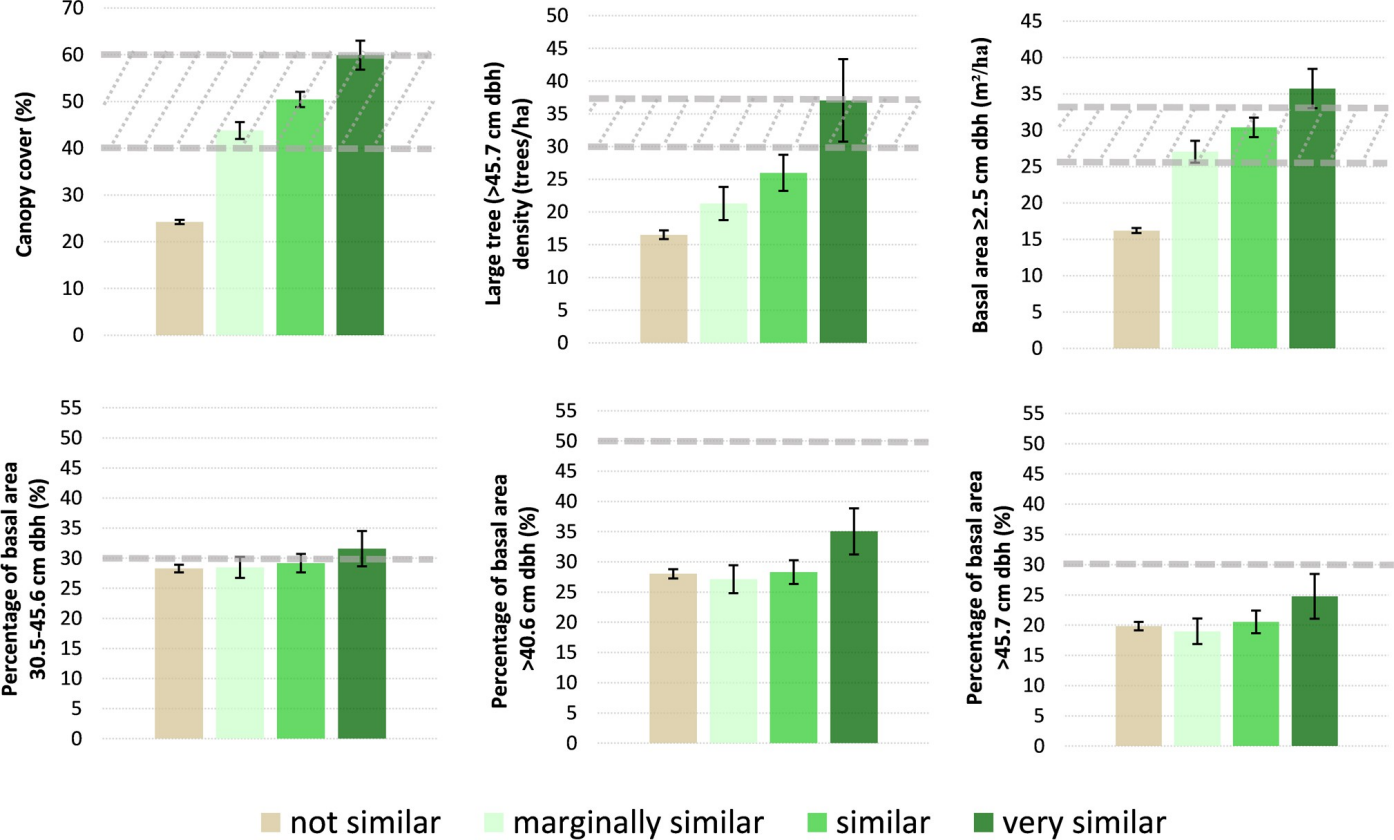
Forest structural attributes of cover type spectral similarity map classes. Dashed gray lines represent minimum desired conditions from Table 1. Height of bars represents mean and 95% confidence intervals are shown in error bars.

Matching FIA plot data to imagery data collected within corresponding years yielded a total sample of 6,414 plots used for inter-class comparison of vegetative structure and species composition. Several structural attributes for the ‘very similar’ map class were consistent with Recovery Plan descriptions of key habitat components of forest types typically used for nesting and roosting and minimum desired conditions (Table 1). Mean canopy cover for this class was 59.9% (95% CI: 56.8, 63.0), large tree density was 42.0 (95% CI: 30.7, 43.3) trees per hectare, and basal area was 35.7 (95% CI: 33.0, 38.4) m^2^/ha. Canopy cover, total basal area, and % basal area from white fir and Douglas-fir all were significantly greater in the ‘very similar’ class than the other three cover type similarity classes, and all increased monotonically from ‘not similar’ to ‘similar’ (Table 2, Fig 3). Large tree density and contribution to basal area from trees > 40.6 cm were also higher in the ‘very similar’ class than the other three classes.

**Table 2:** Results of pairwise comparisons between structural and compositional attributes and similarity class.

	Similarity Class (sample size)					
	not similar (5445)	marginally similar (393)	similar (462)	very similar (114)		
Attributes	means (SD)				F value	*P*
Canopy cover (%)	24.22 (16.99)_2,3,4_	43.80 (18.32)_1,3,4_	50.45 (18.04)_1,2,4_	59.91 (16.90)_1,2,3_	648.52	< .0001
Large (> 45.7 cm) tree density (per ha)	16.52 (24.71)_2,3,4_	21.30 (25.59)_1,4_	25.98 (30.23)_1,4_	37.05 (34.34)_1,2,3_	43.52	< .0001
Total BA ≥ 2.5 cm (m^2^/ha)	16.20 (12.80)_2,3,4_	27.04 (15.26)_1,3,4_	30.39 (14.59)_1,2,4_	35.72 (14.70)_1,2,3_	349.0	< .0001
BA 30.5–45.7 cm (%)	28.29 (23.98)	28.49 (17.76)	29.18 (16.17)	31.59 (15.93)	2.16	0.092
BA > 40.6 cm (%)	27.52 (28.38)_4_	26.85 (23.39)_4_	28.24 (21.50)_4_	35.05 (20.81)_1,2,3_	8.19	< .0001
BA > 45.7 cm (%)	19.84 (25.73)	18.99 (20.49)	20.55 (20.49)	24.75 (20.14)	4.04	0.007
White fir/Douglas-fir BA (%)	1.90 (1.03)_2,3,4_	11.6 (24.5)_1,3,4_	21.9 (32.0)_1,2,4_	43.2 (32.0)_1,2,3_	591.05	< .0001
Pinyon and juniper spp. BA (%)	65.65 (43.56)_2,3,4_	29.65 (35.06)_1,3,4_	12.96 (24.28)_1,2_	3.15 (7.43)_1,2_	476.11	< .0001
Ponderosa pine spp. BA (%)	9.57 (25.21)_2,3,4_	36.22 (37.50)_1,3_	42.17 (36.31)_1,2,4_	31.95 (31.55)_1,3_	465.03	< .0001

Subscripts indicate classes of significant pairwise difference.

**Fig 4:**
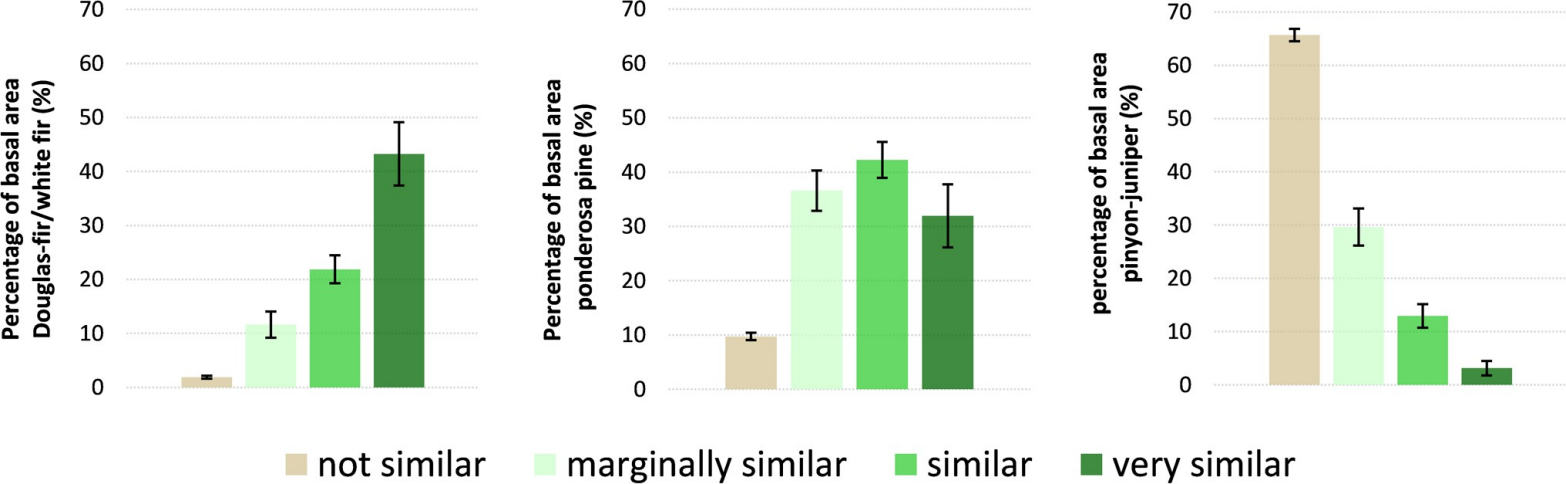
Forest species composition of cover type spectral similarity map classes. Height of bars represents mean and 95% confidence intervals are shown in error bars.

The ‘very similar’ map class had forest species composition similar to desired conditions for nesting and roosting habitat described in the Recovery Plan [15]. This class had a significantly higher percentage (43.2 (95% CI: 37.3, 49.1)) of basal area in Douglas-fir and white fir, the two primary species found in the more mesic mixed-conifer forest type, than other classes, along with a lower percentage of stand basal area (31.9 (95% CI: 26.1, 37.7)) comprised of ponderosa pines and very little pinyon-juniper woodland (3.2 (95% CI: 1.8, 4.6)). The ‘marginally similar’ and ‘similar’ map classes were dominated by ponderosa pine, with the ‘similar’ class having significantly more Douglas-fir and white fir than the ‘marginally similar’ class (Table 2, Fig 4). Pinyon-juniper woodlands dominated the ‘not similar’ class, with very little ponderosa pine or Douglas-fir/white fir contained therein.

**Fig 5:**
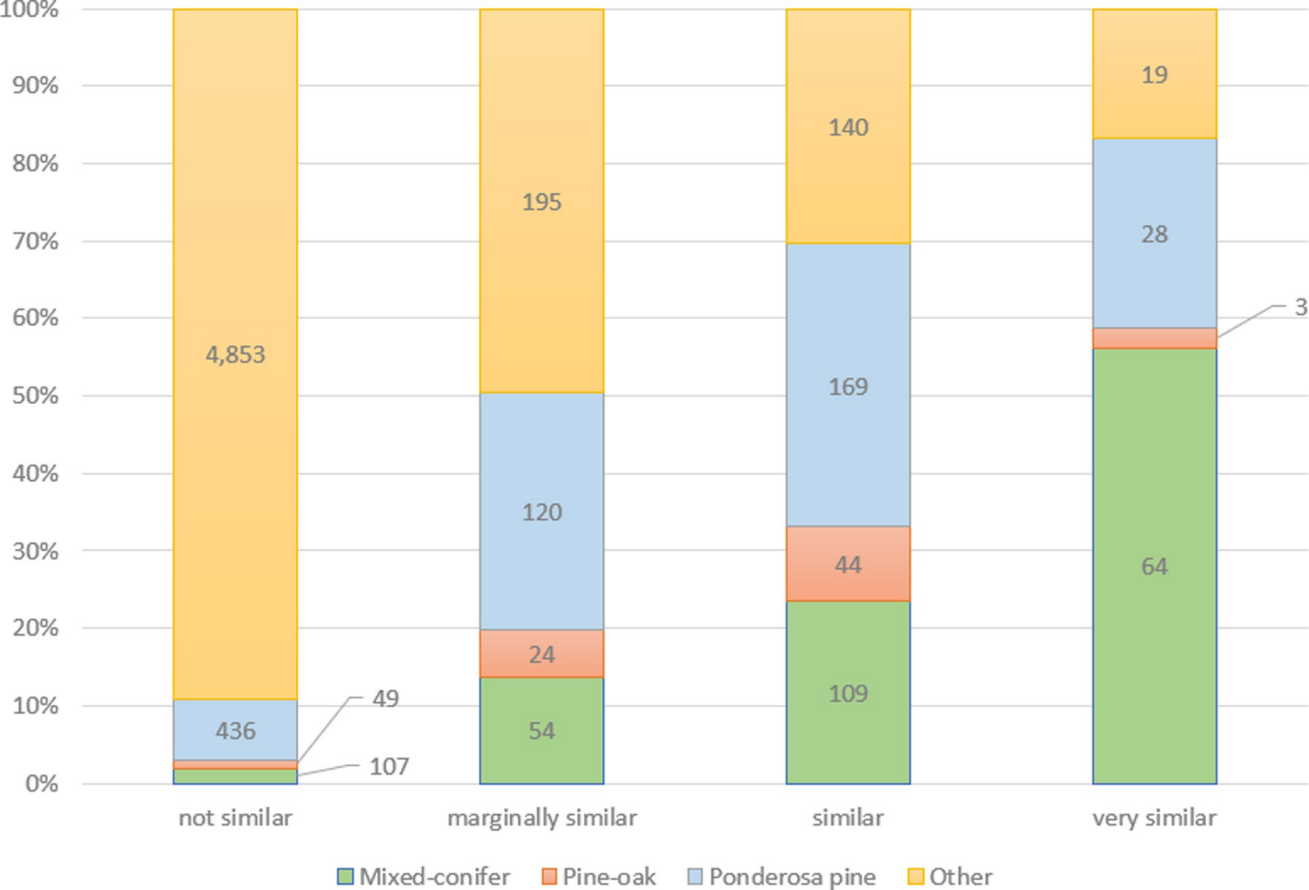
Distribution on FIA plots by forest type and similarity class. Numbers in columns indicate plot count of the specific forest type. Mixed-conifer and pine-oak were defined using descriptions in USFWS 2012. Ponderosa pine and Other were defined by FIA methodologies.

The distribution of FIA plots by forest type varied within similarity classes (Fig 5). The ratio of mixed-conifer plots rose with increasing similarity class while other forest types, consisting mostly of pinyon-juniper, mesquite, and oak woodlands, decreased in the same direction. Mixed conifer plots contributed over half (56%) of plot total in the ‘very similar’ class. Pine-oak and ponderosa pine plots occurred most frequently and provided their greatest contribution to the ‘similar’ class.

### Spatiotemporal trends in amount and distribution of similarity classes

**Fig 6:**
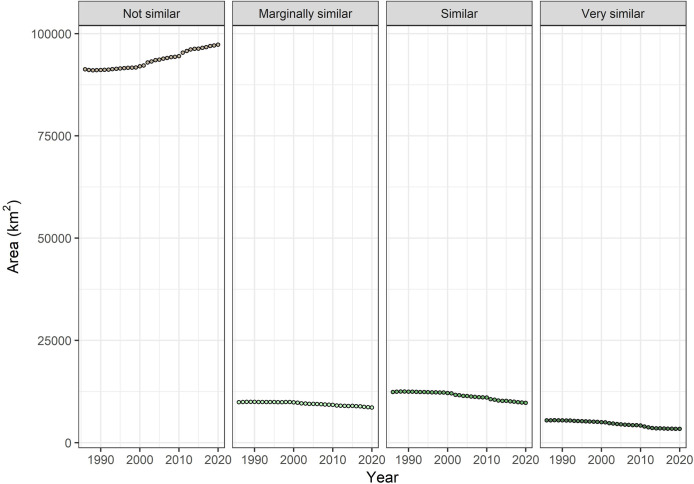
Temporal trends in mapped spectral similarity classes over a 35-year period. Dashed line shows a linear fit over the study period with equation and R^2^ values shown in each panel.

The area in the ‘very similar’ class declined during our 35-year study period, with a mean annual loss of about 61 km^2^ (−1.4%) and a net loss of 2,067 km^2^ (−38%). Amount of area in the ‘similar’ class declined by 2,657 km^2^ (−21.5%) with a mean annual loss of 78 km^2^ (−0.7%). The ‘marginally similar’ class declined by 1,279 km^2^ (−13.0%) with a mean annual loss of 38 km^2^ (−0.4%). The only map class that showed an increase was the class that was ‘not similar’ to nesting/roosting forest cover type. This class increased, on average, about +0.2% each year (177 km^2^) with a net change of +6.6% (6,002 km^2^) over the 35-yr period (Fig 6).

**Fig 7:**
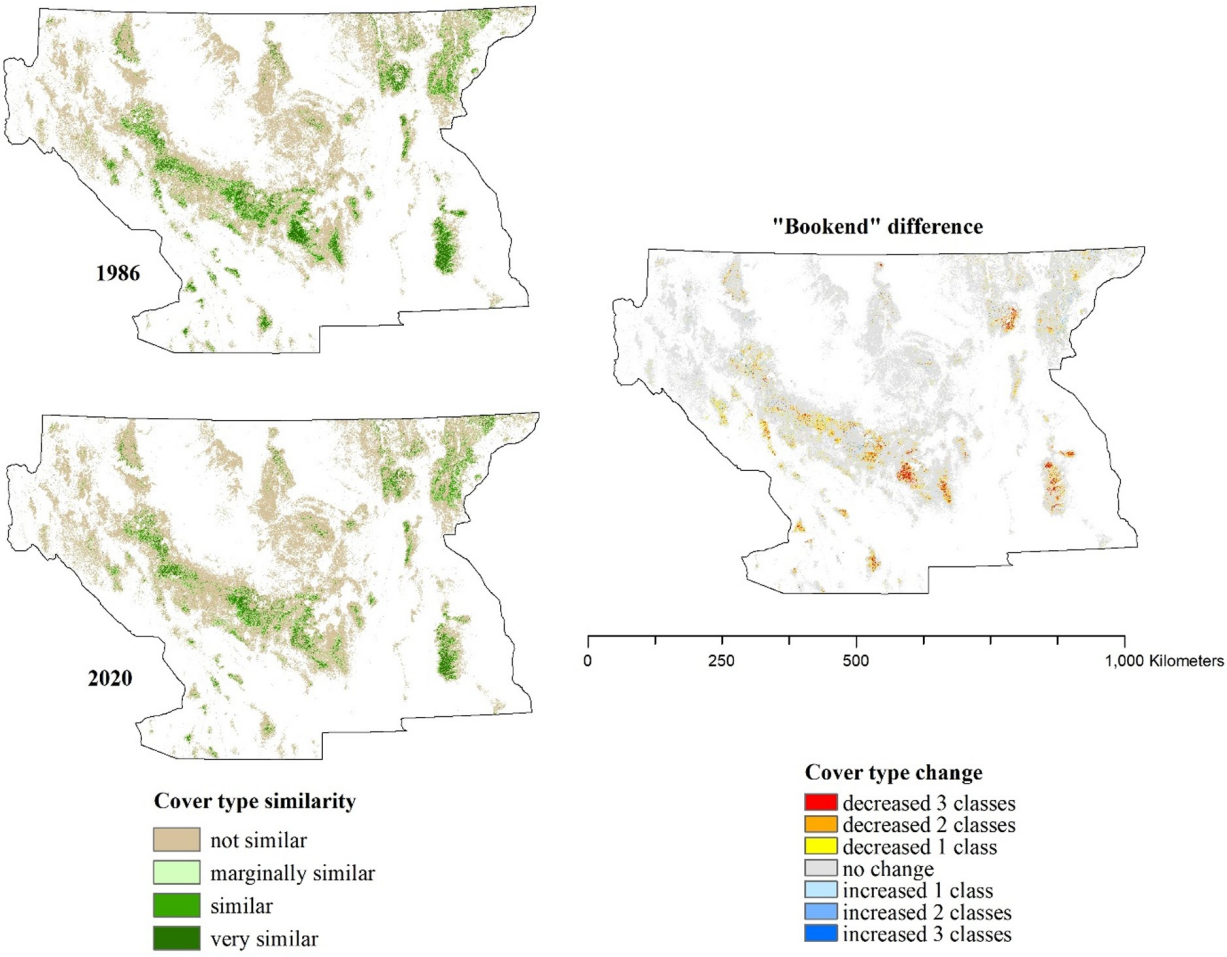
“Bookend” changes in Mexican spotted owl cover type over a 35-year period. The distribution of cover type classes for 1986 and 2020 are shown on the left, and the differenced map showing spatial changes in cover type classes over the study period is shown on the right. Cover type mapping is masked to limits of potential forested land according to the National Land Cover Database.

**Fig 8:**
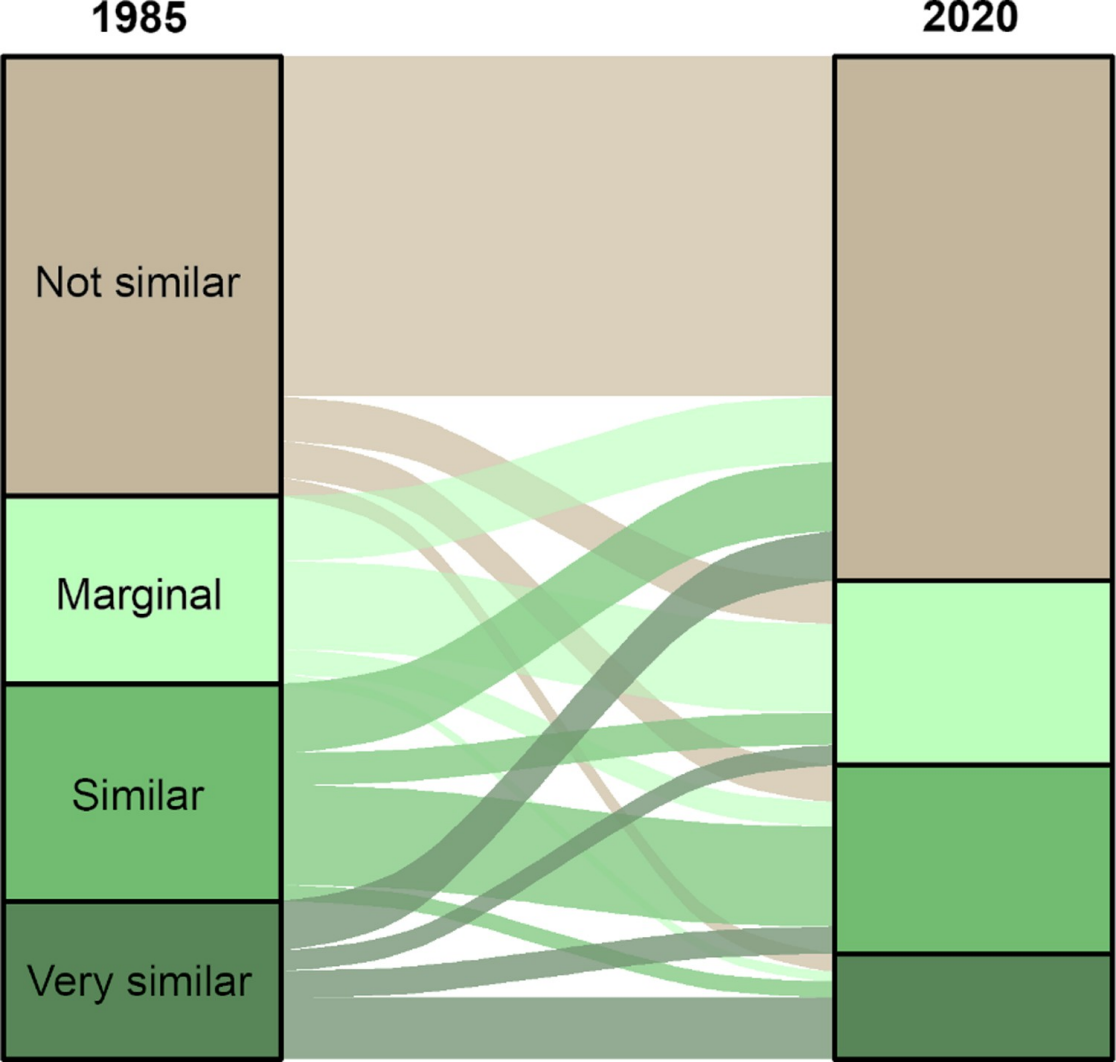
Sankey diagram showing flow of cover type spectral similarity classes between 1985 and 2020. The height of each segment (left and right columns) or flow (connections between columns) is proportional to the total land area in each class. Flows show changes in the spectral similarity class of classified pixels between the two time periods. Values were square root transformed to improve visualization.

**Fig 9:**
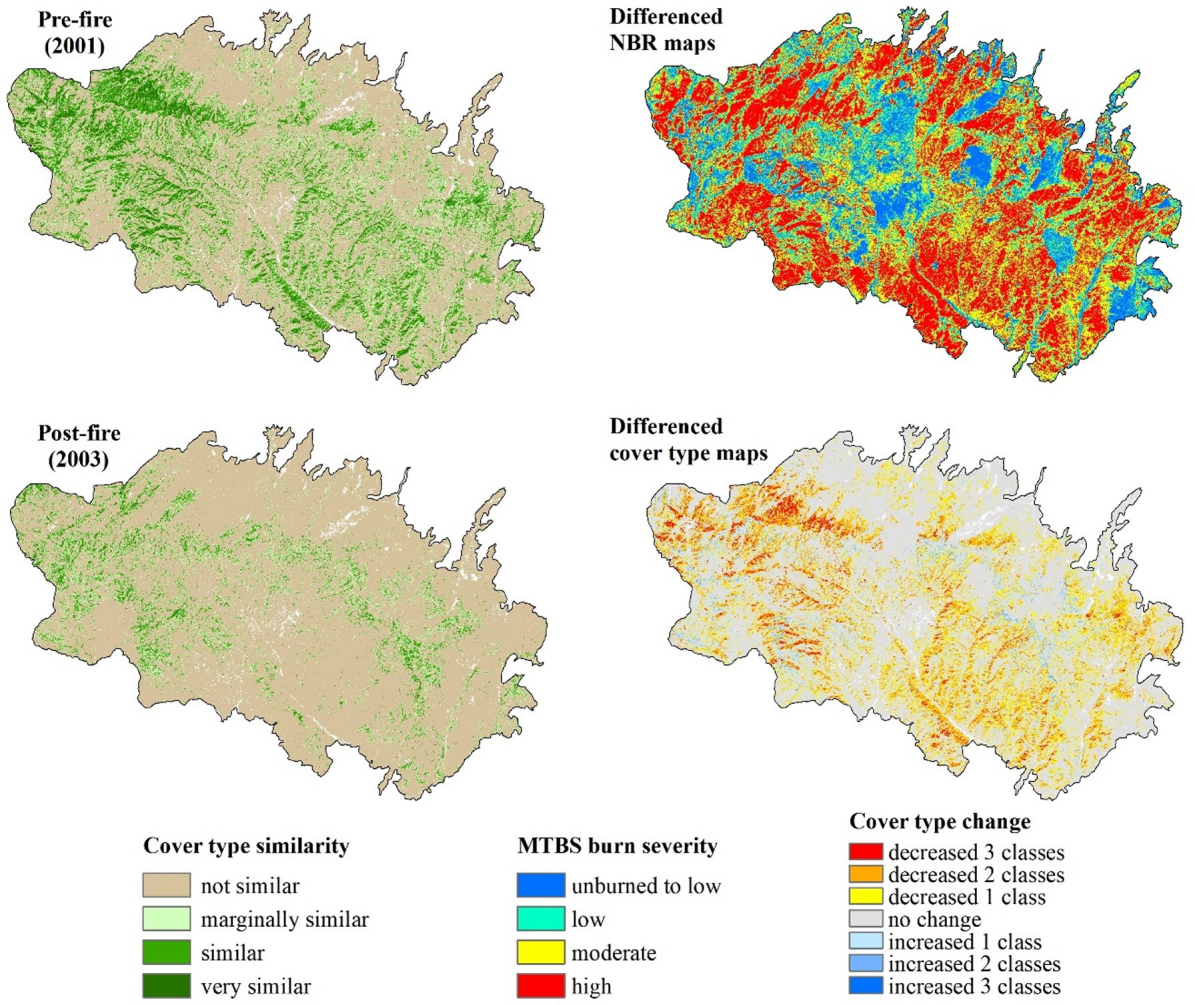
Effects of wildfire on cover type classes. Change detection within the Rodeo-Chediski fire (2002) comparing a MTBS burn severity map (top right) and a differenced cover type map (bottom right).

Differencing maps from the beginning and the ending years or the “book ends” (1986 and 2020, respectively) of this time period allowed us to visualize where forests within the study area have become less similar or more similar to those associated with owl nesting and roosting use (Fig 7) as well as visualize the overall quantitative changes (or “flow”) in area within and among each similarity class over time (Fig 8). We also tested model utility for tracking finer-scale changes related to forest disturbances on the forest vegetation component of owl habitat by focusing on the Rodeo-Chediski fire of 2002, one of the largest recent wildfires within this owl’s range. This fire burned approximately 187,000 ha, of which 68,400 ha (36%) was mapped as high severity, or >75% overstory canopy mortality loss (MTBS; [53]). As a result of the Rodeo-Chediski fire, 83% of forest that was ‘very similar’ (spectrally) prior to the fire became ‘not similar’ after the fire (Fig 9).

## Discussion

Several studies have focused on habitat modeling for the Mexican spotted owl ([15], pgs. 191–193). Although most have focused on nesting and roosting habitat because of its importance in explaining owl distributions [15], they were limited by available technology. We took advantage of advances in computer technology associated with geographic information systems (GIS), species distribution modeling (SDM), processing speed, and cloud computing platforms (e.g., Google Earth Engine) to assess a large scale and temporally rich environmental dataset that we used as predictor variables. The first owl habitat modeling effort used hand-typed timber maps [15]. Yet, more recent efforts have included both remotely sensed biotic data and abiotic variables [54–56]. Here, we have used freely accessible environmental data in a cloud computing platform to model and map important forest vegetation components of owl habitat.

We developed a “ground-truthed” species distribution model by linking Landsat spectral imagery to a robust dataset of Mexican spotted owl nest/roost locations and evaluated fine-scale forest structural conditions across our prediction frame using ground-based stand exam data. The CCDC/Maxent process produced a temporally coherent and accurate time series of maps representing forest vegetation associated with Mexican spotted owl nesting and roosting sites. These maps not only allow for landscape-scale assessments of trends in the amount and spatial distribution of important vegetative components of the owl’s habitat but also provide a basis for robust long-term monitoring. Consequently, by linking map predictions with FIA plot data to estimate areas with high predicted spectral similarity with owl nest/roost areas we successfully modeled the relationship of forest use for a forest-dwelling species solely from spectral signatures of the forest type. Our approach could be extended to other species of interest with highly specific habitat needs provided sufficient location data exist to allow model development.

### Structure and composition of classes similar to owl nest/roost habitat

FIA plot data suggested the forest cover type similarity classes are correlated with structural attributes long considered to be key elements for owl nesting and roosting habitats [57]. However, we did not take into account understory vegetation and down woody material that are important habitat components for owl prey but are not currently mappable at broad spatial and temporal scales using remote sensing. Accordingly, we limit our inferences to forest tree characteristics within owl habitat.

The relative roles of vegetation, topography, and climate in shaping habitat for the Mexican spotted owl vary across its geographical range. For example, vegetation in a recent study accounted for approximately 78% of the variance explained by a multi-scale owl habitat model (with some variance explained jointly with climate and topography) in the Sacramento Mountains but only 46% of explained variance on the Mogollon Plateau [55]. Yet interactions between forest cover, topography, and climate in shaping owl habitat [55] could mean, for example, that two forest stands with similar vegetation structure and Landsat-observed reflectance could have different value to owls within different areas of the species’ range. In addition, the owl is known to nest in steep-walled narrow canyons with lesser amounts of tree cover–taking advantage of rock ledges and caves for nesting. In these areas, forest cover may be less critical for nesting and roosting functions, and spectral signature related to vegetation therefore may be less useful in such areas than in much of our study area.

Both mixed-conifer (dominated by white fir and/or Douglas-fir) and pine-oak forests (comprised primarily of ponderosa pine or Chihuahua pine mixed with mature Gambel oak) were relatively rare across our study area, accounting for 6% and 4% of the total plot count, respectively. However, where mixed-conifer was present, it often provided structure and composition that closely matched what owls are selecting to use. Pine-oak forests were not highly represented in any of our similarity classes though they contributed 10% of the total plot count in the ‘similar’ class. The lack of representation of the pine-oak type in the higher similarity classes might be an artifact of the defining characteristics of pine-oak and/or the ability of our model to capture the specific composition of this forest type. The Recovery Plan definition of the pine-oak forest type requires oak species ≥ 13 cm (drc) contribute ≥ 10% of the stand basal area. Ponderosa pine forests without this requisite oak component (defined simply as a ponderosa pine type) appear to represent much of the forest land in the two higher similarity classes, most notably in the ‘similar’ class. The ponderosa pine forest type often does have an oak component but fails to reach either the basal area contribution or size thresholds to classify them as pine-oak. Our model suggests many ponderosa pine stands–as defined by FIA–could provide nesting and roosting structure that is functionally equivalent to pine-oak stands as defined in the Recovery Plan. Conversely, May et al. (2004) showed that mature oaks within ponderosa pine forest was a key component of nesting sites for owls in northern Arizona [58]. As such, determining whether ponderosa pine stands that do not meet the definitional requirements of pine-oak provide viable breeding habitat needs to be investigated further.

Species distribution models are rarely verified with auxiliary data to validate that areas predicted as ‘suitable’ contain fine-scale elements known to be associated with species presence. The use of FIA plot data to describe our similarity classes relative to forest structure attributes used to guide habitat management was essential for map interpretation. Based on our results, we conclude that our maps of forest cover reflect both structural and compositional features of owl habitat as defined in the Recovery Plan [15], particularly for canopy cover and large tree densities. Since these attributes have been emphasized over basal area attributes for recovery planning [59], the map products produced here should prove useful in guiding such efforts.

The transition from one map class to another followed many pathways related to both forest disturbance and/or forest succession (Fig 8). A stable trajectory was the most common trajectory observed for all map classes (no change in map class between 1986 and 2020). This could be due to lack of disturbances but not enough time for successional processes to push it to the next higher (more similar) map class. The next most common trajectory was a transition to a lower similarity class (excluding the ‘not similar’ class). This trajectory reflected forest disturbances causing a similarity class shift of ≥1 (e.g., ‘very similar’ to ‘not similar’; Fig 9). However, forest succession resulted in slow recruitment of owl nesting/roosting forests because it can take several decades to redevelop after a stand-replacing event [60, 61]. Therefore, we did not expect to see a large amount of transition from the ‘not similar’ class to the ‘very similar’ class, as much of the stand-replacing events occurred relatively late in our temporal window (within 10–15 years). There are also limitations on successional gains in similarity class due to the relative scarcity of mixed-conifer forests. The majority of forested land in our study area is comprised of forest types other than mixed-conifer that are more limited in their potential to provide suitable structure for the owl’s nesting and roosting needs. We did note a slight amount of transitioning from ‘not similar’ to ‘very similar’ occurring in our map products (Fig 8). This might indicate that there is some error and uncertainty in the maps, particularly when it comes to gains in suitable habitat. In this case, a common source of uncertainty in mapping old forests and their growth with remotely sensed data is the effect of canopy shadows [62] as well as resprouting of forb and shrubs that can occur rapidly after a high-severity fire [63]. Our use of CCDC greatly reduced this artifact but did not eliminate it.

### Understanding disturbance effects with classified spectral similarity

**Fig 10:**
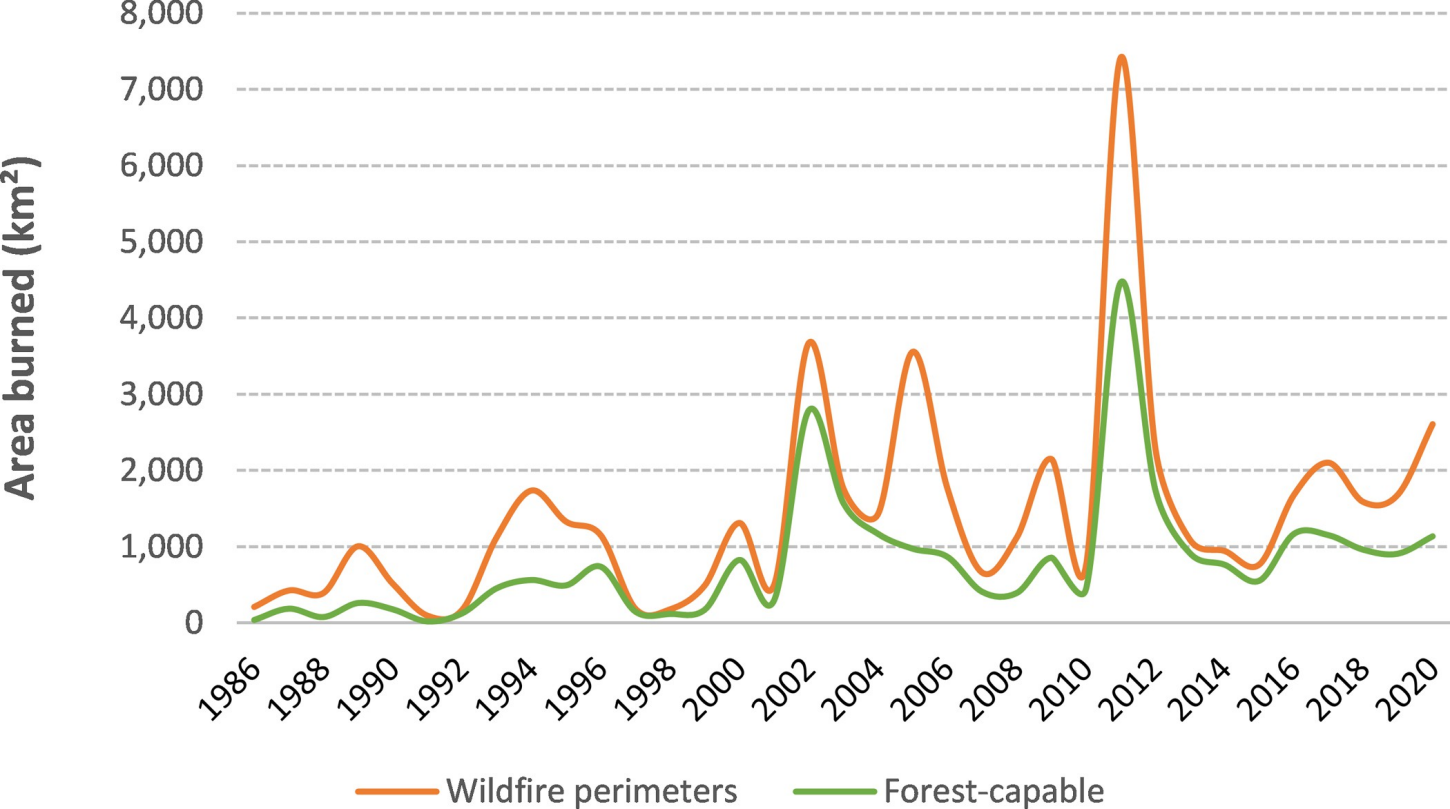
Area burned by large (≥400 ha) wildfires on our study area between 1986 and 2020. The orange line shows total area burned within wildfire perimeters, whereas the green line shows the area within those wildfire perimeters that burned in forest-capable lands.

Much of the reduction we found in the ‘similar’ and ‘very similar’ classes can be attributed to the numerous large wildfires that have occurred in both Arizona and New Mexico during this time. Over the 35-year study period large (≥400 ha) wildfires burned about 5 million hectares within the study area of which 2.8 million hectares were forest-capable. The frequency and extent of these wildfires has increased in the first two decades of this century; 87% of the forested area that burned between 1986–2020 burned after the year 2000 (Fig 10). This increase in wildfire corresponds temporally with marked changes in annual losses of forest classes similar or marginally similar to nesting/roosting forests, noticeable dips in the ‘very similar’ class during the biggest fire years, and a slightly steeper increasing trend in the ‘not similar’ class (Fig 6). This is consistent with results from a portion of the owl’s range that show a departure from historical fire regime with the current fire regime (e.g., fire severity was consistent with the historical expectations but had increased frequency) within mixed-conifer and spruce/fir vegetation types in the southern portion of Basin and Range-West EMU and the southwest portion of Basin and Range-East EMU [64].

Our analysis of cover type change within the Rodeo-Chediski fire was consistent with the findings of an analysis of the Wallow fire, which occurred within our study area and time frame [65]. Using a different model, Wan et al. found that higher fire severity was associated with sharper declines in habitat suitability while fire severity was weakly but significantly positively correlated with pre-fire predicted habitat suitability. Considered in combination, these results suggest owl nesting and roosting habitat might be particularly susceptible to high severity fires, perhaps partly because of its specific structural composition.

At finer spatial scales, our time series of forest cover type similarity maps can be used to better understand the effects of forest disturbance (e.g., wildfire, timber harvesting, etc.) on the vegetation component of owl habitat (Fig 9). Commonly, analyses of fire effects on spotted owls use fire severity maps that are based on differencing relativized normalized burn ratio (NBR) multi-spectral indices [66]. However, the amount of high-severity fire based on NBR can differ considerably from the amount of nesting/roosting forest cover affected by high-severity fire. Therefore, our model can be used alone or in association with NBR-derived maps to gather a more nuanced view of the relationship between fire severity and potential habitat alteration. It also has the potential to evaluate impacts on a scale similar to the Protected Activity Centers (PACs) established around individual owl nest sites [15]. The average size of a PAC is 266 ha and it contains a mix of nesting, roosting, and foraging cover types used by an owl mating pair [15]. Identifying changes in forest cover similarity classes within PAC boundaries that fall within a larger area impacted by wildfire (or other forest disturbances) can provide multiple benefits, such as (1) the ability to quantify changes in PACs individually or in aggregate, which allows managers to stratify for various scales of analysis, such as by ownership, EMU, NBR severity, nest productivity, or forest type; and (2) real effects on vegetation structure (to the degree it is represented by similarity class) within PACs can be gleaned quickly and economically, thus expediting decisions regarding response priorities or post-disturbance prescriptions. Finally, the recovery from disturbance and effectiveness of management actions can be monitored though time, using our tool to help adaptively manage specific areas according to “real time” information.

## Conclusions

Application of our owl-forest vegetation association model to consistently cross-normalized Landsat imagery allowed us to monitor trends and evaluate forest disturbance effects (e.g., wildfire) to forest cover types used by the owl. Likewise, over time, we can measure the slower processes of forest growth and recovery in relationship to this species’ use. Using inventory data to quantify vegetation composition and structure of owl habitat similarly allows us to evaluate trends in those vegetation components, which provide powerful tools to aid recovery efforts for this federally listed species.

One of the delisting criteria defined in the Mexican spotted owl Recovery Plan includes development of a habitat monitoring method that provides a general measure of whether key habitat variables are stable or improving. We used scalable, transparent methods and a cloud-based platform with multispectral and multitemporal imagery to map forest vegetation associated with Mexican spotted owl nesting and roosting habitat over time. In addition, we used publicly available survey data from the FIA program to validate and quantify forest structure and composition within the mapped nesting/roosting forest cover types. Our classification of this index into four distinct cover type classes generally was consistent with known patterns of owl use of mixed-conifer, pine-oak, ponderosa pine, and pinyon-juniper forests and woodlands.

Monitoring of forest vegetation, one component of this species’ habitat, provides insights into the causes and patterns of habitat change from both forest disturbances as well as recovery resulting from forest succession. Understanding these causes and patterns is the next obvious step to inform forest managers about the spatial and temporal dynamics of owl habitat. However, improvements in mapping methods will be needed to address “unrealistic” recruitment of owl forests following a high-severity disturbance that is attributable to artifacts of forb and shrub growth. Finally, the overlay of our habitat vegetation type maps with forest inventory plot data serves not only as way to validate the maps but also as an important step in map interpretation, providing fine-scale details about the structure and composition of stands that are not currently mappable with remote sensing platforms.

We believe much of the change in owl similarity class in our study area was due to fire, but we recognize some of it could have resulted from timber activities, insect outbreaks, and/or disease exasperated by drought. While beyond the scope of this study, the relative impact of these perturbations on owl nesting and roosting habitat can be gleaned using our methodology. If forest disturbances, such as wildfire, continue to accelerate in extent, frequency, and severity in the southwestern US [67], it will be important to have a flexible monitoring program that can be used to quickly update maps so that forest manager’s and regulatory agencies can adapt to changing conditions. Therefore, the methods and results we describe lay the foundation for a long-term habitat monitoring program for this imperiled species.

## Supporting information

S1 Table Summary of data sets and decision rules used by each data set for Mexican spotted owl models. (DOCX)
