# Supplementary material for: Linking robust spatiotemporal datasets to assess and monitor habitat attributes of a threatened species
Source: PLoS One. 2022 Mar 17;17(3):e0265175. doi: 10.1371/journal.pone.0265175 (PMC8929618; doi:10.1371/journal.pone.0265175)
Supplement: S1 Table — (DOCX) [file pone.0265175.s001.docx]

**Supplementary Table 1.** **Summary of data sets and decision rules used by each data set for Mexican spotted owl models.** Types of studies included: demographic studies (D), species recovery planning efforts (R), USFS inventory, monitoring, and project-level surveys (FS), and opportunistic nest/roost observations (O). Step 1 handled data sets with nest and roost locations at the same observation date and territory, step 2 handled data sets with duplicate roost locations on the same observation date, step 3 was a temporal filter for observations during the breeding season, step 4 was a temporal filter for only one nest location per territory per season, and step 5 was a temporal filter for the observation time.

| Data set | Citation(s) | Type | Step 1 | Step 2 | Step 3 | Step 4 | Step 5 | Original *n* | Final *n* |
| --- | --- | --- | --- | --- | --- | --- | --- | --- | --- |
| Seamans/Gutierrez demography | Seamans et al. 1999 | D | x | x | x | x | x | 5963 | 1749 |
| Regional inventory | USFWS 1995 | R/FS |  | x | x |  |  | 874 | 770 |
| RMRS quadrat sampling | N/A | R |  | x | x |  |  | 297 | 134 |
| Bandelier National Monument | N/A | O |  |  | x |  | x | 1 | 1 |
| Cibola National Forest | N/A | FS |  |  | x |  | x | 8 | 8 |
| Los Alamos National Labs | N/A | O |  |  | x |  | x | 2 | 2 |
| Santa Fe National Forest | N/A | FS |  |  | x |  | x | 21 | 18 |
| Coronado National Forest | N/A | FS/O |  | x | x |  | x | 289 | 232 |
| TOTAL |  |  |  |  |  |  |  | **7455** | **2914** |
